# Midventricular Obstructive Hypertrophic Cardiomyopathy during Pregnancy Complicated by Pulmonary Embolism: A Case Report

**DOI:** 10.1155/2012/165918

**Published:** 2012-09-17

**Authors:** Leila Abid, Ahmed Tounsi, Dorra Abid, Mourad Hentati, Samir Kammoun

**Affiliations:** Cardiology Department, Hedi Chaker University Hospital, Sfax 3029, Tunisia

## Abstract

Hypertrophic cardiomyopathy (HCM) with midventricular obstruction (MVO) is a rare condition occurring in 1% of HCM patients. It is characterized by asymmetric left ventricular hypertrophy with MVO and elevated intraventricular pressure gradients. Pulmonary embolism has been associated with mid-ventricular obstructive HCM. Briefly, this case presents an unusual clinical scenario where a young pregnant woman suffering from hypertrophic obstructive cardiomyopathy presents with dyspnea hemodynamic compromise related to pulmonary embolism illustrating hemodynamic challenges created by pregnancy and surgery. We concluded that simple measures such as communication between the cardiology and obstetric teams, understanding of the hemodynamic changes, anesthetic planning, and monitoring were paramount for the success in our patient.

## 1. Introduction

Midventricular obstruction in patients with hypertrophic cardiomyopathy (HCM) is rare, especially in non-Asian populations, occurring in only 1% of HCM patients [1]. It is characterized by asymmetric left ventricular hypertrophy and elevated intraventricular pressure gradients. Pulmonary embolism occurring during pregnancy is a rare accident but it is still responsible for a high mother mortality; it seems to be five to six times more frequent during the pregnancy and the postpartum than for nonparturient women who do not take any estroprogestogens; pulmonary embolism would involve complications for 0.5/1000 pregnancies before delivery. As it presents a lot of diagnostic problems, it is underestimated. Thus, pulmonary embolism complicating a pregnancy in a woman with midventricular obstructive HCM is an exceedingly infrequent clinical presentation. Here, we describe a pregnant woman presenting with a pulmonary embolism who had midventricular obstructive HCM.

## 2. Case Report 

**Figure 1:**
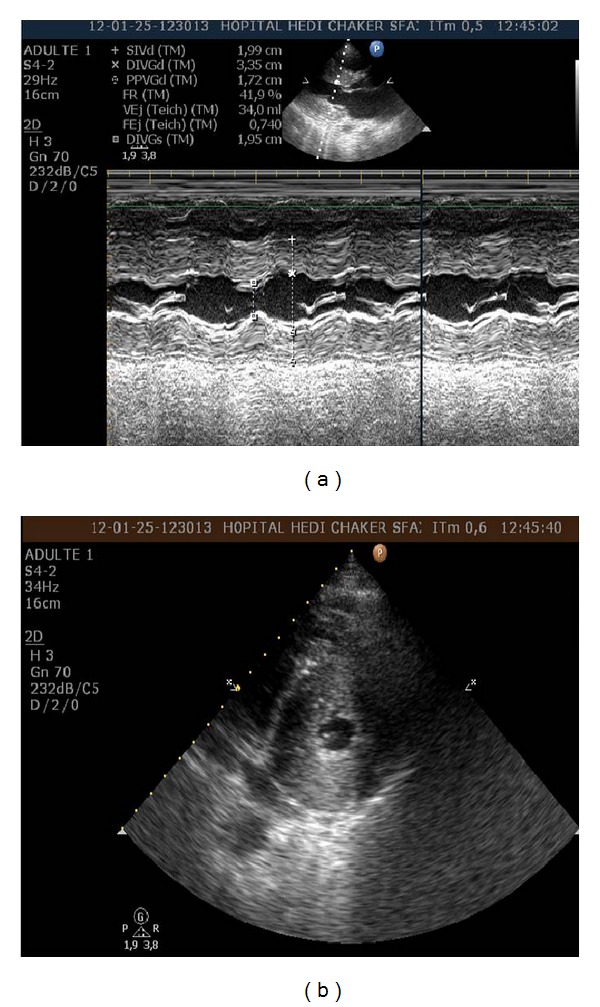
Transthoracic echocardiography revealing inappropriate left ventricular hypertrophy involving the left ventricular (LV) septum (20 mm) and the lateral wall (17 mm), midventricular obstruction.(a)Parasternal long-axis view. (b) LV apical short-axis view.

**Figure 2:**
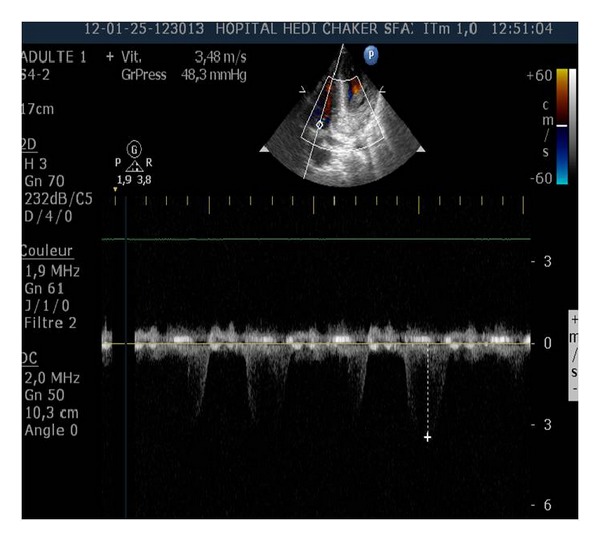
Systolicpulmonary artery pressure (SPAP). Continuous-wave Doppler echocardiography was used to estimate SPAP.

**Figure 3:**
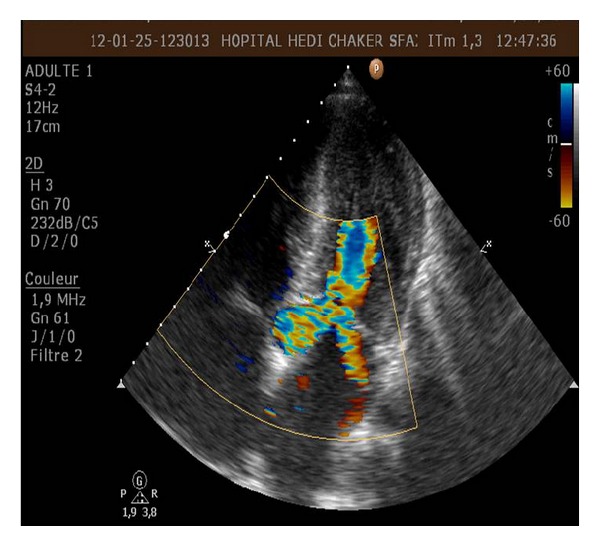
Colour flow Doppler showing evidence of high velocity turbulent flow within the midcavity of the left ventricle.

A thirty-five year old, 34-weeks pregnant woman suffering from hypertrophic obstructive cardiomyopathy diagnosed when she was thirty presented to the emergency room reporting a dyspnea NYHA class IV, chest pain, and heart palpitations. Notably, the family history did not include cardiological disorders or unexpected death due to cardiac dysfunction. The patient was followed for many years. The patient was never severely symptomatic and had a normal tolerance of effort and of controlled physical activity. In particular, she never presented with heart palpitations or dyspnea. She has been treated chronically with metoprolol. The course of the pregnancy remained asymptomatic. Her pulse was 95 bpm. Humeral arterial pressure was about 105/70. An elective systolic murmur mesocardiac point, accentuated by the valsalva manoeuvre, was auscultated. There were no signs of jugular turgor. Her respiratory rate was 18 breaths per minute with 98% oxygen saturation on room air; there were no pulmonary pathological noises. Electrocardiography revealed sinus tachycardia, left ventricular hypertrophy by voltage criteria, and ST segment depressions with T wave inversions in the lateral leads. A chest radiograph was normal contrasting with the severity of dyspnea. Arterial blood gas values were PH = 7.51, PaO_2_ = 89 mmHg, and PaCO_2_ = 27 mmHg. She was anemic at the time of admission (hemoglobin = 8.9 g/dL). Other laboratory values were white blood cell count 12.48 ∗ 10^3^ mL, platelet count 135 ∗ 10^3^/mL, calcium 9.6 mg/dL, potassium 4.0 mEq/L, and creatinine 0.63 mg/dL. Two-dimensional echocardiography demonstrated an aortic root of normal diameter (30 mm), an aortic tricuspid valve with normal systolic spread, a left atrium with slightly increased dimension (30 cm^2^), a left ventricle with normal dimension and preserved systolic function (the ejection fraction was 65%), and a marked concentric hypertrophy of the left ventricule with midventricular systolic obliteration (intraventricular septum 20 mm, posterior wall 17 mm). The right atrium and right ventricle had normal dimensions, with normal right ventricular function. An elevated right heart pressure was observed. Moderate tricuspid regurgitation was recorded (Figures 1(a) and 1(b)). Right ventricular systolic pressure was calculated to be 60 mmHg (Figure 2). Continuous Doppler showed the presence of a mean midventricular gradient of 53 mm Hg and a peak gradient of 125 mmHg, a peak velocity of 5.5 m/s, and the presence of relaxation diastolic dysfunction (Figure 3). In summary, the echocardiographic exam showed severe pulmonary hypertension, hypertrophic obstructive cardiomyopathy with a very hypertrophic left ventricle with normal internal dimension and global systolic function. Pulmonary embolism was suspected because of very high pulmonary hypertension and lungperfusionscintigraphywasperformed andshowedadefect of infusionof4 and 8segmentsof the right lung. Venous echo Doppler of lower limb was normal. Therapy with heparin was started and we decided to perform a caesarian under general anesthesia to give better haemodynamic control was recommended because of the elevated risk of heart failure and arrhythmias: Therapy with heparin was started and we decided to perform a caesarian under general anesthesia because of the elevated risk of heart failure and arrhythmias. At the anaesthesiological examination the patient was classified as anaesthesiological risk ASA IV and informed of the elevated anaesthesiological risk. The postoperative course remained stable. The systolic arterial pressure was about 100–115 mmHg and SpO_2_ remained stable about 99%. The new-born child was male, 2725 g in weight, 46 cm in length, and presented with Apgar indices of 6 and 9 at 1 and 5 min from birth, respectively. The postoperative course of the patient was regular, asymptomatic, and haemodynamically stable for the whole period of stay in the intensive care unit. The electrocardiographic monitoring showed periods of monomorphic ventricular tachycardia, with a maximal cardiac frequency of 150 heart beats. The patient was kept under observation in the postintensive care unit for 2 days before being discharged and retransferred to cardiology, where she continued her therapy with enoxaparin (6000 UI twice a day) and Bisoprolol (10 mg/day). The patient was discharged from the hospital a week after delivery. At followup of 12 months after birth, the child exhibited normal growth and development for his age and did not show any clinical abnormalities and the patient did not suffer from any symptoms. Midventricular peak gradient decreased from 125 mmHg to 54 mmHg during 4 months followup.

## 3. Discussion

Midventricular obstruction occurs in only 1% of HCM patients [1]. Associated with mutations of the essential or regulatory light chains of the myosin heavy chain, this obstruction is characterized by asymmetric left ventricular hypertrophy and elevated interventricular pressure gradients. In midventricular obstructive HCM, echocardiography reveals hypertrophy only in the mid portion of the left ventricle and hypertrophied papillary muscles, resulting in a systolic obstruction of the midventricle. Continuous-wave Doppler echocardiography reveals high flow velocities with an abnormally high intraventricular pressure gradient across the obstruction. Consequently, a paradoxical jet flows from the apex toward the base during left ventricular isovolumetric relaxation and the early diastolic filling period. No paradoxical jet was noticed in this case. Pregnant patients with HC have special considerations due to the unique cardiovascular changes inherent to pregnancy. In later stages, aortocaval compression or higher blood loss during labor can reduce preload considerably. On the other hand, pain and labor stress can generate a sympathetic stimulation, increasing the heart rate and contractility, contributing for hemodynamic deterioration. The importance of pulmonary embolism in pregnancy cannot be exaggerated. In developed countries where women no longer die from sepsis and hemorrhage, it is the leading cause of maternal mortality [2]. The management of pulmonary embolism in pregnancy is difficult because few internists have much experience of managing pregnant women; and few obstetricians have any experience of pulmonary embolus. In addition there is confusion about the safety of maternal investigations for the fetus, particularly when these investigations involve ionizing radiation and also confusion about the effects that any maternal therapy may have on fetal well being. Plethysmography has shown that veins are more distensible in pregnancy and recent Doppler studies indicate that lower limb blood flow is reduced in pregnancy [3]. These effects are likely to be due to a combination of hormonal influences on the structure of vessel walls and obstruction to blood flow caused by the enlarging uterus. It has been known for some time that the composition of the blood changes in pregnancy probably because of high estrogen levels. For example the levels of clotting factors II, VII, VIII, X, and fibrin all increase in pregnancy [4]; to a certain extent, this is offset by an increase in thrombolysis [5]. However, recent interest in natural anticoagulants has shown that pregnancy also decreases some of their activities: specifically, activated protein C resistance is increased (decreased activated protein C resistance ratio) even in the absence of Factor V Leiden [6] and protein S levels also decrease [7]. So it is likely that changes in the composition of the blood in pregnancy relate to both an increase in clotting factors and a decrease in natural anticoagulation. Appropriate care of a pregnant patient with suspected PE demands prompt diagnosis with a reliable imaging study. Ridge et al. performed an audit of pregnant patients with suspected PE that showed lung scintigraphy produces diagnostic images more frequently than does pulmonary CTA, most nondiagnostic CTA studies showing an artifact due to transient interruption of contrast material by unopacified blood from the IVC [8]. The management of midventricular obstructive hypertrophic cardiomyopathy is uncertain, difficult, and complicated by high incidences of fatal ventricular arrhythmias and sudden cardiac death [9]. The value of beta blockers, which are the first choice for treating subaortic HCM, has not been established in midventricular obstructive HCM. Recently, Hamada et al. concluded that the class Ia antiarrhythmic, cibenzoline can attenuate left ventricular pressure gradients and ameliorate left ventricular dysfunction in patients with HCM caused by a midventricular obstruction [10]. Dual-chamber pacing and percutaneous septal ablation have been proposed as an alternative to surgery, such as myectomy, but the long-term benefits procedural safeties of these options are unknown [11]. We think that our case is original because the combination of PE complicating obstructive HCM in pregnancy is seldom described in the literature. There was only one case reported by Paranskaya et al. [12] which dealt with dynamic left ventricular outflow tract obstruction in pulmonary embolism. And it is not the same thing because our patient has an organic obstructive hypertrophic cardiomyopathy and pulmonary embolism was a complication during pregnancy. The diagnosis and management of pulmonary embolism in pregnancy is difficult, especially when it is associated to a dyspnea which can be linked to other diseases such us obstructive hypertrophic cardiomyopathy. Severe pulmonary hypertension and normal chest radiography contrasting with the severity of the dyspnea in pregnant woman with HCM should make us think of pulmonary embolism. The treatment must be emergent with a collegial decision combining anesthetic and cardiologic opinions. The misdiagnose can lead to a delay of an adequate treatment and so to a catastrophic event for both the baby and the mother.
